# Biological behavior exploration of a paclitaxel-eluting poly-l-lactide-coated Mg–Zn–Y–Nd alloy intestinal stent *in vivo*

**DOI:** 10.1039/c9ra10156j

**Published:** 2020-04-16

**Authors:** Zhanhui Wang, Zongbin Sun, Baowei Han, Qiuxia Zheng, Shaopeng Liu, Bingbing Zhang, Tinghe Duan

**Affiliations:** Department of Surgery, Luoyang Central Hospital Affiliated to Zhengzhou University 288 Zhongzhou Road Luoyang 471000 China zhanhuiwangxyjt@163.com +86 379 6389 2095 +86 379 6389 2095; The Second Affiliated Hospital of Zhengzhou University Zhengzhou 450003 China

## Abstract

As a new type of intestinal stent, the MAO/PLLA/paclitaxel/Mg–Zn–Y–Nd alloy stent has shown good degradability, although its biocompatibility *in vitro* and *in vivo* has not been investigated in detail. In this study, its *in vivo* biocompatibility was evaluated by animal study. New Zealand white rabbits were implanted with degradable intestinal Mg–Zn–Y–Nd alloy stents that were exposed to different treatments. Stent degradation behavior was observed both macroscopically and using a scanning electron microscope (SEM). Energy dispersion spectrum (EDS) and histological observations were performed to investigate stent biological safety. Macroscopic analysis showed that the MAO/PLLA/paclitaxel/Mg–Zn–Y–Nd stents could not be located 12 days after implantation. SEM observations showed that corrosion degree of the MAO/PLLA/paclitaxel/Mg–Zn–Y–Nd stents implanted in rabbits was significantly lower than that in the PLLA/Mg–Zn–Y–Nd stent group. Both histopathological testing and serological analysis of *in vivo* biocompatibility demonstrated that the MAO/PLLA/paclitaxel/Mg–Zn–Y–Nd alloy stents could significantly inhibit intestinal tissue proliferation compared to the PLLA/Mg–Zn–Y–Nd alloy stents, thus providing the basis for designing excellent biodegradable drug stents.

## Abstract 2

Mg–Zn–Y–Nd alloy stents coated with MAO/PLLA/paclitaxel coating were implanted into the New Zealand rabbits intestine to investigate the biocompatibility and degradation behavior.

## Graphical abstract

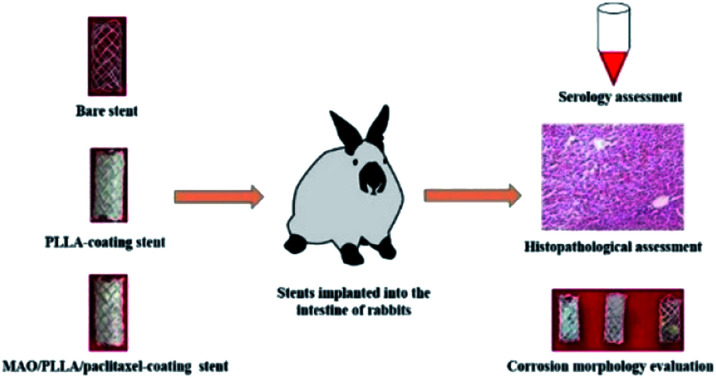

## 1. Introduction

Bowel obstruction due to intestinal stricture formation is a well-known complication of enteral diseases, including malignant and benign strictures. Factors causing benign intestinal obstructions include Crohn's disease and anastomotic stenosis.^1^ The recommended treatment for strictures involves self-expanding metal or plastic stents. However, the application of these stents is related to several common problems, including new stricture formation, perforation, migration, tissue ingrowths, and repetitive endoscopy.^2–6^ To avoid the complications of permanent metal and plastic stents, biodegradable polymer alloy stents have recently been introduced.

Biodegradable stents (BD_S_) made of magnesium alloys demonstrate superior performance to their polymeric counterparts due to excellent mechanical properties similar to SS316L stainless steel, which cannot be achieved by polymers.^7^ Some studies have shown that magnesium alloys can be used as orthopedic implants or cardiovascular stents.^8–15^ However, it is well known that magnesium alloys will gradually corrode under natural conditions. And the major limitation of biodegradable magnesium alloy stents is their low corrosion resistance. To lengthen the degradation time, alloying is used as one of the methods to enhance corrosion resistance and mechanical strength. Moreover, scaffold surface polymer coating is also important for mechanical strength.^16^ Degradable polymers, such as PLLA, PLGA, have good plasticity, mechanical properties, and biocompatibility, which are most commonly used biomaterials for surface modification of alloys to enhance the corrosion resistance of alloys.^17–21^ Therefore, PLLA is considered as a coating material to improve the corrosion resistance of magnesium alloy surface in this paper. At present, there are many ways to improve the corrosion resistance of the alloy surface, besides polymer coating, micro-arc oxidation (MAO) process is also one of the surface modification technologies of magnesium alloys. At high breakdown voltage, various processes such as electrochemical, thermodynamic and plasma chemical reactions occur, accompanied by spark discharge to produce thicker, harder and wear-resistant ceramic coatings that can effectively improve the corrosion resistance of the alloy surface.^22,23^ However, to reduce neointimal growth, biodegradable magnesium alloy stents can be coated with polymers containing antiproliferative drugs. Even though previous research report refered to that the clinical efficacy of paclitaxel was affected by drug resistance, in view of its excellent antiproliferation property in the treaty of breast cancer paclitaxel was regarded as one of the effective antiproliferative drugs.^24^ At the same time, it has been reported in literatures that both paclitaxel and sirolimus can effectively inhibit neointimal hyperplasia of coronary artery within a month dose range, and the effect has a certain degree of safety.^24–27^ It can effectively reduce the degree of vascular restenosis. Therefore, paclitaxel and sirolimus are commonly used in drug-eluting stents for the treatment of coronary stenosis. However, whether paclitaxel that can effectively inhibit the occurrence of vascular endothelialization, as well as the biological safety of the paclitaxel-eluting intestinal stent to important organs and tissues of human body, are unknown. Therefore, paclitaxel is selected as a sustained-release drug of the stent to explore the compatibility of the drug-eluting stent in intestinal environment in this study. However, few studies to date have reported on the biocompatible and biodegradable properties of magnesium alloy in the intestine.

Mg–Zn–Y–Nd alloys have excellent biodegradability and biocompatibility, so they have been used as biomaterials for cardiovascular stent research.^28^ In this paper, we selected Mg–Zn–Y–Nd alloys as the alloy material of degradable intestinal drug-eluting stents. The biocompatibility *in vitro* was evaluated by the cytotoxicity test. In addition, animal experiments are carried out by placing scaffolds in different treatment groups. We evaluated the *in vivo* biocompatibility of a paclitaxel-eluting poly-l-lactide-coated Mg–Zn–Y–Nd alloy applied as a biodegradable intestinal stent through animal study.

## 2. Experiment

### 2.1 Materials preparation

The paclitaxel-eluting poly-l-lactide-coated Mg–Zn–Y–Nd alloy stents used in this research were 25.0 mm in length, with a 10.0 mm diameter. A new Mg–Zn–Y–Nd alloy was prepared by induction in a low carbon steel crucible at about 740 °C in the carbon dioxide/sulphur hexafluoride atmosphere (volume fraction of 3000 : 1) using high purity magnesium, high purity zinc, and magnesium-25nd (99.97 wt%) and magnesium-25y (wt%) master alloys.^28^ Magnesium alloy stent fibers were fabricated using a single screw extruder. PLLA ((C_6_H_8_O_4_)_*n*_, intrinsic viscosity: 3.4 dl g^−1^ (CHCL_3_/25 °C)) and paclitaxel were of reagent grade and purchased from the Science and Technology Company of Solarbio (Beijing, China) and the Academy of Pharmaceutical Sciences (Jinan, China). In this study MAO/PLLA and MAO/PLLA/paclitaxel coatings were prepared using the dip coating method. The PLLA was dissolved in dichloromethane with a proportion of 0.03 g ml^−1^. Paclitaxel (>98% content) was dissolved in a PLLA solution with a concentration of 0.008 g ml^−1^. Because paclitaxel has higher drug loading accuracy in the concentration range of 5 mg ml^−1^ to 15 mg ml^−1^.^29^ Mg–Zn–Y–Nd alloy stents were immersed in the solution for 30 min, pulled with a dip coating method, and evaporated for 0.5 h. The process was repeated according to thickness requirements and the final product placed in a vacuum-drying oven to remove the solvent for 24 h. In this study, the thickness of PLLA coating is about 15.1 μm ± 3.1. Before the *in vivo* experiments, all samples were sterilized with 29 kGy of cobalt-60 radiation. And PLLA coated magnesium alloy specimens and PLLA/paclitaxel coated magnesium alloy specimens were also prepared by dip coating method as the above. The control stent was identical, except for the absence of paclitaxel and poly-l-lactide.

In this study, some wafer samples were treated with MAO to improve the corrosion resistance. The composition of the electrolyte used for MAO was listed in Table 1. When all reagents are dissolved, deionized water was used to determine the volume and mix evenly. High frequency single pulse power supply (ys9000d-300-40) was used in micro arc oxidation process. The alloy stent was used as the anode to connect the power supply, and the stainless steel plate was used as the cathode to connect the power supply. At a constant voltage, the voltage increases from 0 V to 260 V at a rate of 1.6 V s^−1^. The samples were treated for 20 minutes, and then washed with deionized water and dried naturally.

**Table 1:** The chemical composition of MAO

Reagent	Na_3_PO_4_·12H_2_O	NaOH	C_3_H_8_O_3_
Amount (L^−1^)	54 g	2 g	6 g

### 2.2 Animal model

Animal experiments were performed according to the Guidelines for the Nursing and Use of Laboratory Animals and approved by the Ethics Committee of Luoyang Central Hospital Affiliated with Zhengzhou University. Forty-eight clean adult New Zealand white rabbits with an average body weight of 2000 g (±121 g) were randomly divided into four groups. In the first group, rabbits did not receive any intestinal implants and were assigned to the negative control group. In the second group of 12 rabbits, the Mg–Zn–Y–Nd alloy stents were implanted into the intestine. This group was named the Mg–Zn–Y–Nd alloy stent group. In the third group of 12 rabbits, the PLLA/Mg–Zn–Y–Nd alloy stents were implanted into the intestine. In the last group of 12 rabbits, the MAO/PLLA/paclitaxel/Mg–Zn–Y–Nd alloy stents were implanted into the intestine. The operation was performed under intraperitoneal anesthesia with 3% pelltobarbitalum natricum (2 ml kg^−1^). The skin was incised layer-by-layer and a 10 mm intestinal incision was made parallel to the length–diameter of the intestine. The Mg–Zn–Y–Nd alloy stents were embedded in the intestinal incision and the abdomen was sutured layer-by-layer. After the operation, about 10 ml of sodium chloride were administered to the rabbits. The rabbits were able to conduct regular activities following recovery of consciousness, including eating and drinking.

### 2.3 Methods

#### 2.3.1 Cell toxicity assay for paclitaxel loaded in the stent

Indirect contact cytotoxicity test was performed to prove indirectly that paclitaxel is loaded in the stent designed in this study. Pure DMEM culture medium containing 10% fetal bovine serum (FBS) was used as the negative control and DMEM containing 0.64% phenol was the positive control. NCM-460 cells (human colonic epithelial cells) were suspended in complete medium. Cell concentration was adjusted at 1.0 × 10^5^/ml, inoculated in 96 well plates. The cells were cultured in the incubator (37 °C) for adherence. The incubator was maintained at 37 °C and 5% CO_2_, and the extraction medium was replaced every 24 h. NCM-460 cells were cultured in different concentrations of extract medium for 72 h, respectively. To indirectly determine the cytotoxicity of magnesium alloy extracts. Whether paclitaxel was loaded into magnesium alloy scaffolds was judged indirectly by observing the cell proliferation of different treatment groups.

#### 2.3.2 Macroscopic and SEM analysis

Magnesium alloy stents implanted in the intestinal tracts of New Zealand white rabbits were removed at different time points (2, 5, 8, and 12 days). First, the overall structure and corrosion morphology of the stents were observed macroscopically. Scanning electron microscopy (SEM) was used to analyze the corrosion morphology of every stent group (bare body, PLLA/Mg–Zn–Y–Nd, and MAO/PLLA/paclitaxel/Mg–Zn–Y–Nd alloy scaffolds). Energy dispersion spectrum (EDS) was used to evaluate the corrosion degree of magnesium alloy stents.

#### 2.3.3 Biocompatibility analysis of *in vivo* study

Before stent removal, 3 ml of blood from every rabbit in the MAO/PLLA/paclitaxel/Mg–Zn–Y–Nd alloy stent group were taken for serological analysis of liver and kidney functions to assess whether systemic inflammation was induced. In this study, hematoxylin–eosin (HE) was used for tissue staining. The intestinal tissues around the scaffold, including heart and liver organs, were fixed with a 10% formalin solution for 24 h. The sections were then embedded in paraffin. The heart and liver tissues were stained with HE only. Intestinal tissue paraffin sections were incubated with the Bcl-2 (diluted to 1 : 200) and Bax (diluted to 1 : 200) antibodies. Elivision immunohistochemical technique was used. Finally, the stained sections were observed using an optical microscope and the number of positive cells in each visual field were recorded.

## 3. Statistical analysis

The differences between the two groups were assessed using one-way ANOVA. Step-wise regression analyses were conducted to evaluate the dose effects. The values were considered significant when *p* < 0.05. Statistical values are shown in the relevant experiments.

## 4. Results

### 4.1 Cell toxicity assay

**Fig. 1:**
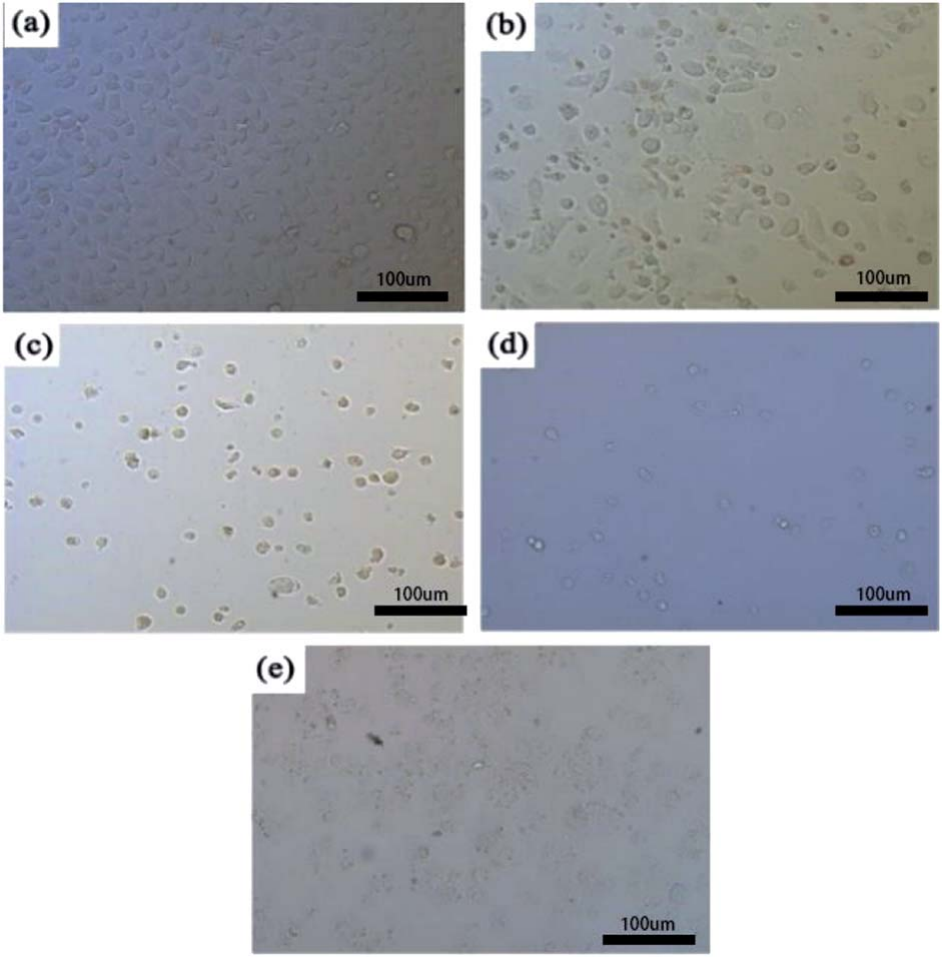
Cell morphology of 8 NCM-460 cells cultured in PLLA/paclitaxel/Mg–Zn–Y–Nd alloy extract at different concentrations for 72 hours: (a) negative control (DMEM medium containing 10% fetal bovine serum), (b) 30% concentration, (c) 60% concentration, (d) 100% concentration, (e) positive control (DMEM containing 0.64% phenol).

Fig. 1 showed the morphology of NCM-460 cells cultured in different concentrations of extracts for 72 hours. As can be seen from Fig. 1, the cells in the negative group were normal and healthy, and there were more active cells. However, with the increase of extract concentration, the number of cells decreased gradually, and the cell morphology was abnormal. Compared with the other two groups, the number of cells in the paclitaxel-coated group was significantly reduced and the cell morphology was abnormal. Paclitaxel has the effect of inhibiting cell proliferation. Through the cytotoxicity test, it can be confirmed that paclitaxel has been successfully loaded into magnesium alloy stents.

### 4.2 Assessment of macroscopic corrosion morphology in intestinal drug-eluting stents

**Fig. 2:**
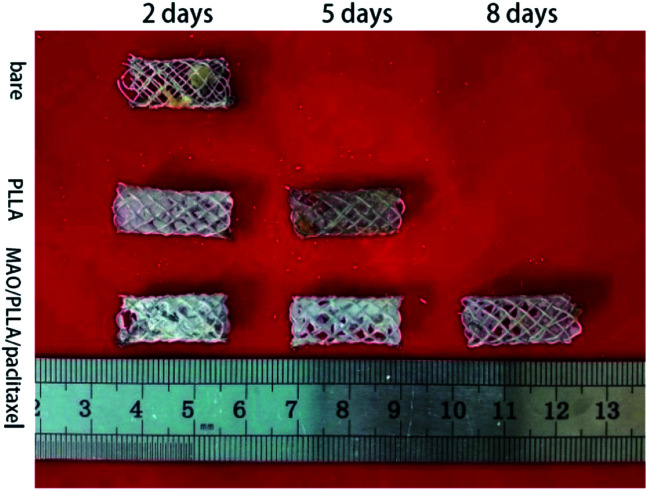
Macromorphology of Mg–Zn–Y–Nd alloy stents for different treatment groups implanted in intestine of New Zealand rabbits for 2, 5, 8 and 12 days.

Magnesium alloy intestinal stents from different treatment groups were implanted into New Zealand rabbits for 2 days and a small amount of degradation products was attached to the surface of the stents. Fig. 2 demonstrates that the structure of the PLLA-coated magnesium alloy stent was still intact five days after implantation into rabbits. It can also be observed that a small amount of fibers began to break on the stent of the PLLA-coated magnesium alloy group and that the amount of degradation products attached to the surface increased. However, no magnesium alloy stent was found in the rabbit intestinal tract in the bare stent group after implantation for 5 days. After stent implantation for 8 days, only the MAO/PLLA/paclitaxel-coated Mg–Zn–Y–Nd stents were found in the rabbits. No stents were found in the other two groups. Moreover, no magnesium alloy stents were found in the three groups after implantation into the rabbit intestinal tract for 12 days.

### 4.3 SEM corrosion morphology assessment in intestinal drug-eluting stents

**Fig. 3:**
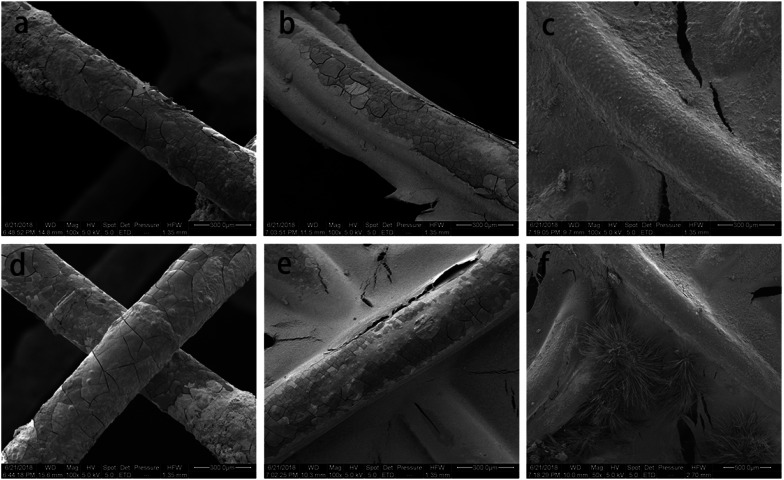
The corrosion morphology observed by SEM of magnesium alloy filaments for different groups of alloy stents after implantation in New Zealand white rabbits for two days: (a and d) bare magnesium alloy stents, (b and e) PLLA coated magnesium alloy stents, (c and f) MAO/PLLA/paclitaxel coated magnesium alloy stents, (a–c) stents located at the unconnected position, (d–f) stents located at the joint.

Fig. 3 represents the local corrosion morphology of magnesium alloy stent fibers implanted for 2 days. A few cracks and flaking formed on the surface of the silks, while the stent structure for uncoated Mg–Zn–Y–Nd alloy remained intact (Fig. 3a and d). The number of cracks and debris adhesions to the surface of scaffold threads in the PLLA-coated stent group was much smaller than that in the exposed group. However, surface of the scaffold threads in the MAO/PLLA/paclitaxel-coated stent group was smoother than that of bare Mg–Zn–Y–Nd alloy stents, showing better resistance to corrosion compared to that of bare Mg–Zn–Y–Nd alloy stents (Fig. 3c and f). The surfaces of the threads at the end of the bracket was rougher than that inside the stent and there were more small cracks (Fig. 3).

### 4.4 EDS spectra corrosion morphology assessment in intestinal drug-eluting stents

**Fig. 4:**
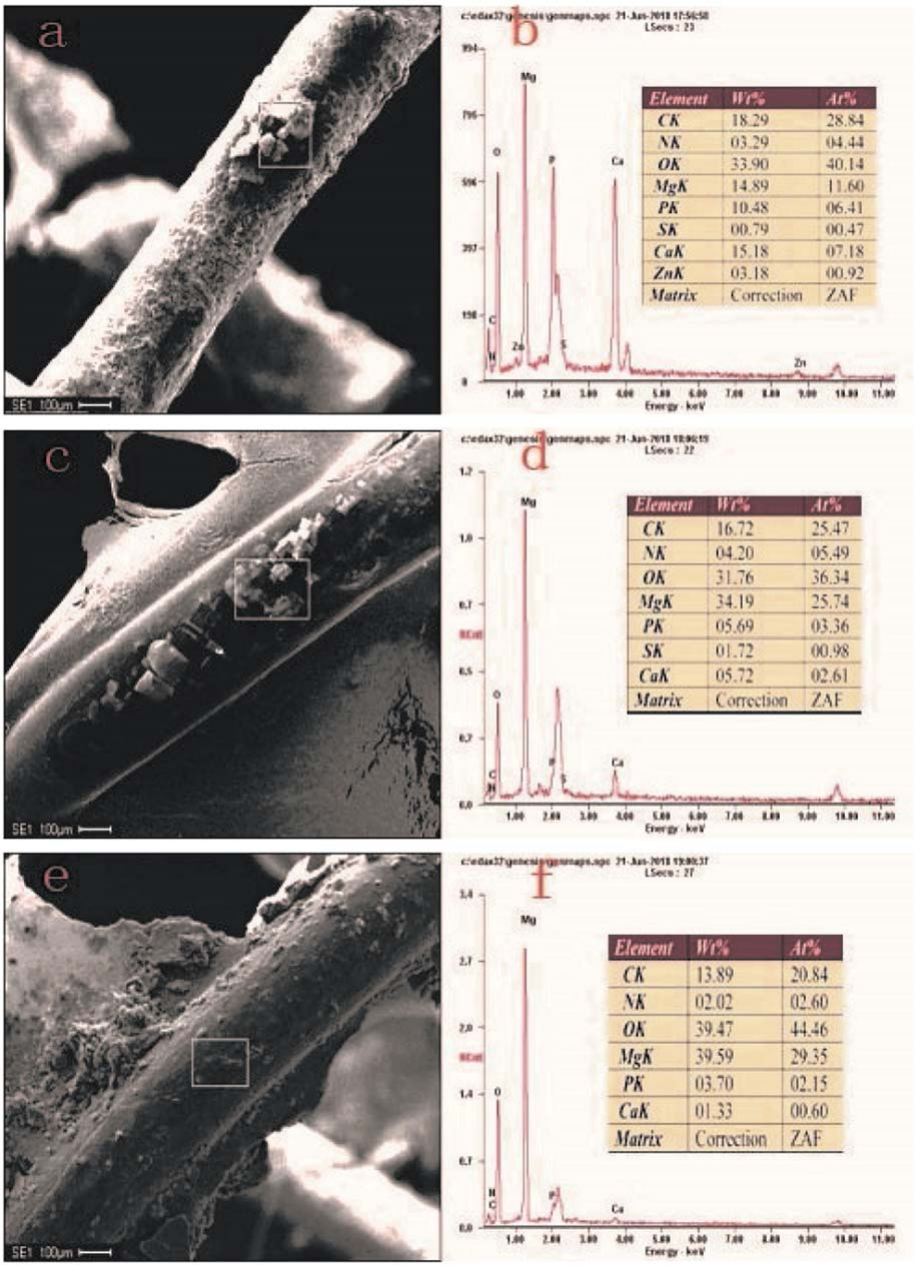
The corrosion morphology and EDS spectrum of magnesium alloy stent in the intestine of New Zealand white rabbits in different treatment groups two days after implantation: (a and b) bare stents, (c and d) PLLA coated stents, (e and f) MAO/PLLA/paclitaxel coated stents.

SEM and EDS results 2 days after implantation of stents from different treatment groups are represented in Fig. 4. A lot of degradation products were present on the surface of bare scaffold filaments (Fig. 4a), where the content of P and Ca was 10.48% and 15.18%, respectively (Fig. 4b). Gray-white degradation products were present on the surface of the PLLA-coated stent filaments (Fig. 4c), with the content of P and Ca of 5.69% and 5.72%, respectively (Fig. 4d). A small amount of degradation products was present on the surface of the MAO/PLLA/paclitaxel coated scaffolds (Fig. 4e), with the content of P and Ca of 3.70% and 1.33%, respectively (Fig. 4f). These results showed that degradation products contained some insoluble inorganic substances such as magnesium carbonate and calcium phosphate. Moreover, the content of P and Ca in the MAO/PLLA/paclitaxel-coated stent group was significantly smaller than that in the other two groups (*p* < 0.05), while the contents of P and Ca in the PLLA-coated stent group was significantly smaller than that in the bare stent group (*p* < 0.05). EDS analysis showed that the MAO/PLLA/paclitaxel-coated stent group had better corrosion resistance than the other two groups.

### 4.5 Histopathological assessment of important organs

**Fig. 5:**
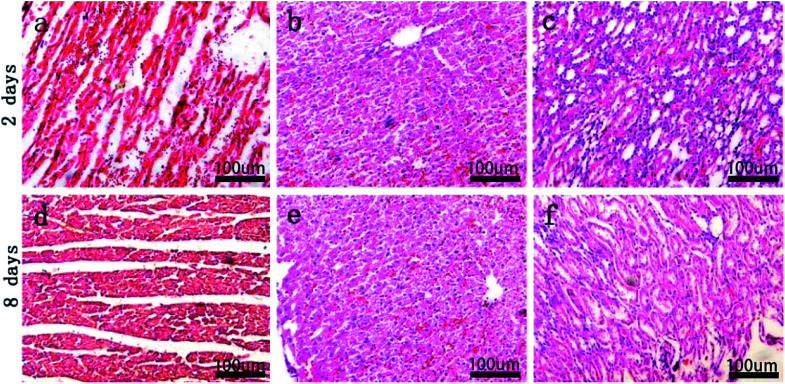
Pathological images of important organs in healthy rabbits after implantation of MAO/PLLA/paclitaxel coated Mg–Zn–Y–Nd alloy stents for 2 days, 8 days: (a and d) cardiac tissue, (b and e) liver tissue, (c and f) kidney tissue.

Pathological assessment images of important rabbit organs after implantation of the MAO/PLLA/paclitaxel-coated Mg–Zn–Y–Nd alloy stents at 2 and 8 days were shown in Fig. 5. Myocardial fiber morphology was presented in Fig. 5a and d, with no obvious abnormality of nuclear structure and no infiltration of inflammatory cells in the interstitial cells found at each stage. There was no obvious abnormality in the morphology of hepatocytes, as evident from the structure of hepatic lobules, with no infiltration of inflammatory cells between hepatocytes at different time points (Fig. 5). The morphology and structure of renal corpuscles, tubules, and collecting ducts were not abnormal. This indicated that the MAO/PLLA/paclitaxel-coated Mg–Zn–Y–Nd alloy scaffolds had good biocompatibility.

### 4.6 Histopathological evaluation of intestinal tissues around stents

**Fig. 6:**
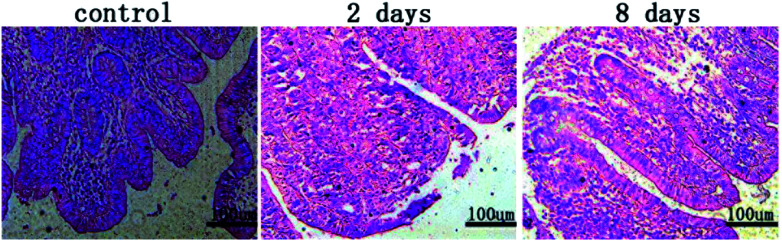
The intestinal histopathological sections of rabbits implanted with alloy stent at different times: (a) section of the control group, (b) section at 2 days after implantation, (c) section at 8 days after implantation.

**Fig. 7:**
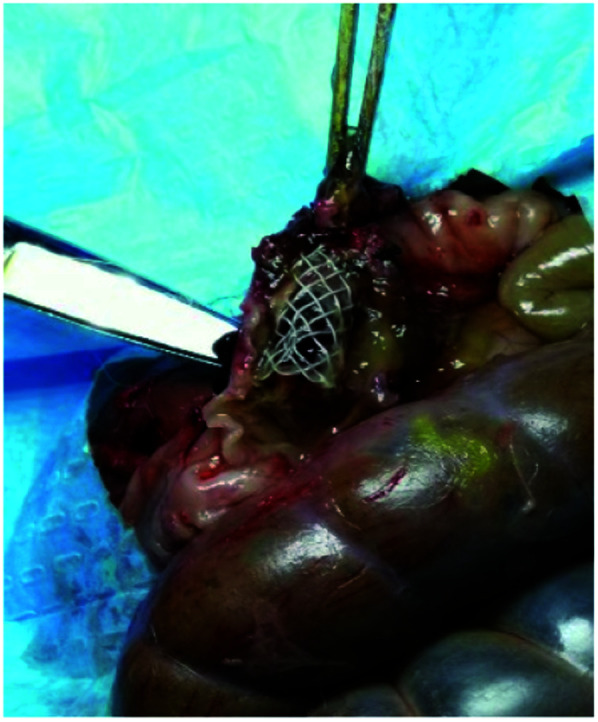
Picture when the stent was removed after implanted into the rabbits for a period of time.

Pathological sections revealed the effect of stent implantation on surrounding intestinal tissue in rabbits at different stages (Fig. 6). And Fig. 7 showed the relationship between the stent and the surrounding intestinal tissue when the stent was removed after implanted into the rabbits for a period of time. Two days after stent implantation, the intestinal mucosa around the MAO/PLLA/paclitaxel-coated magnesium alloy intestinal stent showed a small amount of damage and exfoliation, with a certain amount of inflammatory cells infiltrating the sample tissue. Eight days after stent implantation, the stripped area of intestinal mucosa around the stent was significantly reduced compared to 2 days after implantation. Inflammatory cell infiltration was also obviously reduced. The intestinal mucosa of the stripped area began to grow again, intestinal epithelial proliferation was inhibited, and tissue damage tended to recover, as shown in Fig. 6.

### 4.7 Immunohistochemical evaluation of intestinal tissues around stents

**Fig. 8:**
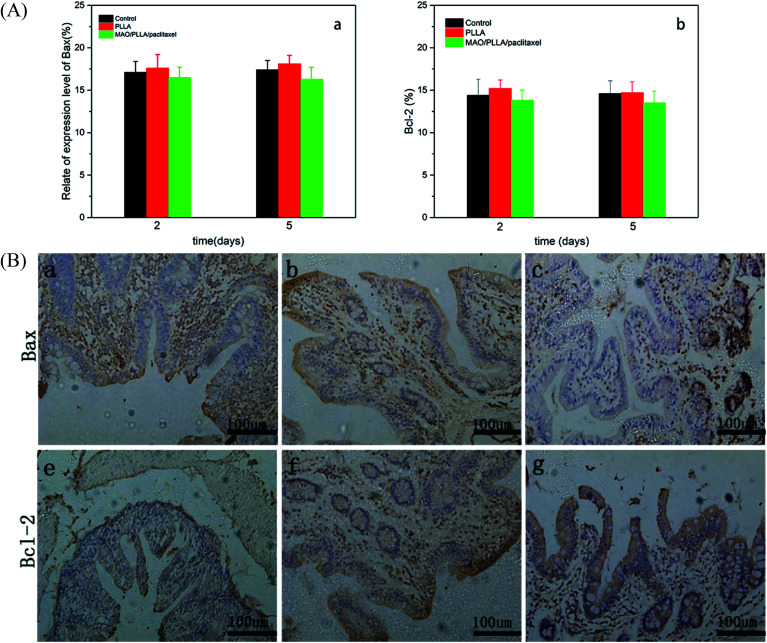
The histogram represents the expression level of two antibodies and immunohistochemical section: (a and e) section of the control group, (b and f) section at 2 days after implantation, (c and g) section at 5 days after implantation.

Immunohistochemical staining results for Bax and Bcl-2 were shown in Fig. 8. The antibody expression levels for Bax and Bcl-2 from immunohistochemical results for intestinal tissue around the stents were shown in Fig. 8A(a and b). Two days after the operation, the expression level of Bax in the MAO/PLLA/paclitaxel stent group was lower than that in the PLLA-coated magnesium alloy stent group and slightly lower than that in the control group, although no statistically significant differences were present (*p* > 0.05). The expression of Bax in the PLLA group increased slightly 5 days after the operation compared to that 2 days after the operation, and was slightly higher than that in the control group with no statistically significant differences (*p* > 0.05). The expression level of Bcl-2 in the MAO/PLLA/paclitaxel group was slightly lower than that in the PLLA stent and control groups during the whole experimental period (*p* < 0.05). The expression level of Bcl-2 in the PLLA group was slightly higher on the fifth day than on the second day (*p* < 0.05). However, there was no significant difference in Bcl-2 expression between the PLLA and control groups (*p* > 0.05).

### 4.8 Serological assessment of rabbit liver and kidney function

The changes in liver serum and kidney function after 8 days of implantation of the MAO/PLLA/paclitaxel-coated Mg–Zn–Y–Nd alloy intestinal stents in rabbits were shown in Table 2. The level of alanine aminotransferase (ALT/GPT) after implantation was slightly higher than that before the experiment in only one rabbit and slightly lower in the remaining two rabbits. Similarly, the aspartate aminotransferase (AST/GOT) levels decreased slightly in only one rabbit and increased slightly in the remaining two rabbits. Among the indicators of renal function 8 days after implantation of the MAO/PLLA/paclitaxel-coated magnesium alloy stent into the rabbit intestinal tract, creatinine (Cre) levels in two rabbits were slightly higher than those before the stent implantation, while urea levels in one rabbit were slightly higher than those before the stent implantation. Although serological liver and kidney function indices of some rabbits after stent implantation were higher than those before stent implantation, the increase was not significant and the overall change was maintained in the normal range.

**Table 2:** Serological index content of liver function and renal function

		Index of liver function (mmol L^−1^)		Index of renal function (mmol L^−1^)	
		ALT/GPT	AST/GOT	Cre/Cre−	UREA
Control	M1	107	121	44	5.6
	M2	112	118	52	4.5
	M3	114	132	49	5.4
8 days	M1	116	106	42	4.4
	M2	107	120	56	3.6
	M3	109	138	53	5.7

## 5. Discussion

Intestinal stenosis is a common surgical disease. It is also a complication of some diseases or surgical operations, including intestinal ulcers, benign and malignant intestinal tumors, Crohn's disease, ulcerative colitis, intestinal tuberculosis, and abdominal surgery, among others. Endoscopy is an important way to treat intestinal stricture, especially colon or rectal stricture. The stent is not only used for the treatment of malignant intestinal obstructions, such as colon cancer obstruction, but also for the treatment of benign intestinal obstructions, including colon and anastomotic fistulae, perforation, and inflammatory obstructions. Currently, stents for intestinal stenosis include self-expanding metal stents (SEMS), self-expanding plastic stents (SEPS), and BDS.^30,31^ Since 1990, there have been many reports of stent placement for the treatment of advanced obstructive colon cancer showing good success rates, with few complications, including 3.76% perforation, 11.8% stent displacement, 7.34% restenosis, and 0.58% cumulative mortality.^32^ In eight studies with a total of 199 patients Thomas *et al.* found that the migration rate of self-expanding removable stents was 26.4%, with an average of 17 days. Within 4–8 weeks, stent removal and perforation rates were 87% and 1.5%, respectively.^33^ Although metal stents have good clinical effects in the treatment of intestinal stenosis, their clinical application causes several common problems, including new stenosis formation and intimal tissue remodeling.^1^ Therefore, prevention of intestinal stenosis, good biodegradability, and biosafety are very important for the clinical application of stents.

### 5.1 Biocompatibility of intestinal eluting stents

Magnesium ion is a degradation product of magnesium alloy and an important element of human metabolism. Moreover, it is the fourth largest cation in human plasma.^34^ The content of magnesium in bone is about two-thirds of the total amount of magnesium in the human body. The other one-third is found in tissue and about 1–2% of magnesium is in the extracellular fluid.^35^ In addition, magnesium ions play an important role in regulating the homeostasis of human body. They can inhibit the release of calcium from sarcoplasmic reticulum in response to a sudden influx of extracellular calcium.^36^ Early typical symptoms, such as anorexia, nausea, vomiting, and sleepiness, occur when magnesium is deficient.^37^ Research has shown that drinking natural mineral water rich in magnesium sulfate seems to be a first-line solution to functional constipation before starting medication.^38^ Di *et al.* used degradable magnesium alloy Mg–1Sr, a new compound, to study its biocompatibility *in vivo* in New Zealand white rabbits.^39^ Histological studies did not reveal physiological abnormalities or diseases. It has been reported that appropriate amounts of zinc are very important for regulating the immune response. Zinc supplementation has a potential effect on immune function impairment caused by a decrease in serum zinc concentration due to advanced age.^40^ Without affecting the normal healing process, the main challenge of stenting in the treatment of coronary heart disease is to prevent restenosis.^41^ Therefore, stent design with good compatibility and prevention of intestinal stricture is critical for the treatment of intestinal stricture.

In this study, it was proved that paclitaxel had been successfully loaded into magnesium alloy stents through the cytotoxicity test. After the MAO/PLLA/paclitaxel/Mg–Zn–Y–Nd alloy stents were implanted into rabbits in this study, no significant abnormalities were found in the pathological analysis images of the heart, liver, and kidneys. In addition, serological indicators of liver and kidney function in rabbits showed no significant abnormalities before and after stent implantation. This may be related to the fact that the stent does not affect normal metabolism of magnesium ions in rabbits. Xu *et al.* prepared organic coatings with PLLA on pure Mg substrate by the spin-coating method, and the research demonstrated that PLLA films could significantly improve SaOS-2 cell cytocompatibility of the alloy.^17^ There are also previous reports that excessive magnesium ion concentration or alkaline stress could produce negative stimuli for the cell population.^42^ Moreover, low magnesium diet may lead to higher intra-cellular ratio of Ca : Mg, leading to hypertension and insulin resistance.^43^ A precious study revealed that although low magnesium intake is related to constipation, high doses of oral magnesium have a defecation effect.^44^ This effect of magnesium may be related to the following facts: they are absorbed from the intestinal cavity and play a penetrating role to maintain water, thereby increasing the fluidity of the lumen content.^45^

Paclitaxel is a natural compound, which is isolated from the Pacific Redwood. Paclitaxel reacts with β-tubulin in microtubule to induce microtubule polymerization, which leads to cell proliferation stopping at G2/M phase and finally result in tumor cell apoptosis.^46^ Previous studies have shown that stent implantation can cause the formation of endothelialization in the surrounding tissue.^47–49^ Although the time of stent implantation in intestinal tract is short, there may not be obvious tissue proliferation around the stent. However, the number of epithelial cells and fibroblasts in intestinal mucosa tissue is increased in the process of intestinal endothelialization.^49^ While the antibody used in immunohistochemistry can be detected by the detection of cell proliferation and expression of related factors were used to indirectly explore the compatibility of stent on intestinal tissue. In this study, histopathological examination showed that the MAO/PLLA/paclitaxel/Mg–Zn–Y–Nd and PLLA/Mg–Zn–Y–Nd alloy scaffolds did not induce systemic inflammation (*p* > 0.05), while immunohistochemical results showed that degradation products of the MAO/PLLA/paclitaxel/Mg–Zn–Y–Nd alloy scaffolds significantly inhibited proliferation of intestinal cells. The PLLA/Mg–Zn–Y–Nd scaffold degradation products did not induce significant proliferation of intestinal tissue cells (especially epithelial cells) (*p* > 0.05). These results suggest that short-term implantation of the MAO/PLLA/paclitaxel/Mg–Zn–Y–Nd alloy scaffolds can significantly inhibit the endothelialization of intestinal epithelial cells. The PLLA/Mg–Zn–Y–Nd alloy stents were not conducive to the formation of intestinal endothelialization. It has been reported that the use of SEPS can cause low epithelial proliferation and make the stents easy to remove.^50^ In a study of drug-loaded cardiovascular stents, it was found that proliferation of rat vascular smooth muscle cells (SMCs) was successfully inhibited when paclitaxel was released from the poly(carbonate urethane)urea coating.^51^ Stephen *et al.* found that a magnesium-based drug delivery system had a stronger long-term inhibitory effect on the proliferation of SMCs cultured *in vitro* compared to stainless steel, which may be related to the degradation of magnesium alloy matrix greatly accelerating and improving the pharmacokinetics of drug release *in vitro*.^52^

In this study, five rabbits were found to have stents displaced during their removal. No other organ damage, perforation, or obstruction were found. Geiger *et al.* analyzed 26 original articles, where 63 patients received long-term treatment with SEMS. Severe complications were found, including bladder perforation, ileostomy, massive hemorrhage, and obstruction.^53^ The clinical data for self-expanding metal colon stent implantation in some hospital from January 1996 to May 2012 were retrospectively analyzed. The surgical technique success rate was 92.26% (*n* = 441), with clinical success rate of 78.45% (*n* = 375), and complication rate during follow-up of 18.5%. The incidence of complications with stainless steel stents was higher than that with nickel–titanium alloy stents.^54^

### 5.2 Degradation characteristics of intestinal eluting stents in a rabbit model

It is well known that traditional medical and scaffolding materials such as stainless steel and titanium alloy are non-absorbable. Previous studies have found that long-term retention of these non-absorbable materials in the human body can cause damage to human health.^55^ Therefore, magnesium alloy scaffolds with biodegradable properties are of great significance for the treatment of intestinal diseases. Magnesium-based substrates easily react in aqueous media in the following manner:^56^

1 Mg(s) + 2H_2_O(aq) ⇋ Mg(OH)_2_(s) + H_2_(g)↑

2 Mg(OH)_2_(s) ⇋ Mg^2+^(aq) + 2OH^−^(aq)

Hydrogen and insoluble magnesium hydroxide are produced in this reaction, which directly changes the concentration of magnesium ion, pH, and other biochemical conditions. However, these changes may have some impact on human health.

In this study, macroscopic corrosion morphology of Mg–Zn–Y–Nd alloy intestinal stents implanted into New Zealand white rabbits showed that bare stents can be located only after 2 days of implantation. PLLA-coated stents could not be found 8 days after implantation. The MAO/PLLA/paclitaxel-coated stents were intact at 2, 5, and 8 days, and no longer present in the intestinal tract at 12 days. This phenomenon may be related to the stent structure collapse caused by stent degradation after 8 days of implantation in rabbits and its eventual removal from the intestine. The bare body scaffold completely degraded 5 days after implantation, most likely because the scaffold without a protective layer comes into direct contact with the intestinal environment and has poor corrosion resistance, thus accelerating scaffold degradation. In the PLLA/Mg–Zn–Y–Nd alloy scaffold group, a small amount of fibers on the scaffold began to break, while the amount of surface degradation products increased. However, the PLLA-coated magnesium stents were not found in the rabbit intestines after 8 and 12 days of stent implantation. This phenomenon indicates that the MAO/PLLA/paclitaxel/Mg–Zn–Y–Nd alloy scaffolds have stronger corrosion resistance than the PLLA/Mg–Zn–Y–Nd alloy scaffolds. SEM analysis indicated that the microwire surface of the MAO/PLLA/paclitaxel scaffolds is more complete and cleaner than the other two groups, as shown in Fig. 3. EDS results in Fig. 4 showed that the content of P and Ca in the MAO/PLLA/paclitaxel stent group is significantly lower than that in the other two groups, which is related to better corrosion resistance of the MAO/PLLA/paclitaxel stents in intestinal environment and fewer corrosion products. In conclusion, these results suggest that stents treated with the MAO/PLLA/paclitaxel coating can significantly improve corrosion resistance of the magnesium alloy. Previous studies have confirmed that the PLLA-coated alloy surface can significantly delay alloy degradation rate, thereby improving corrosion resistance of alloy materials.^52,57^ Application of micro-arc oxidation can significantly enhance corrosion resistance of biodegradable implants.^58^

In clinical application, tissue self-healing usually takes 2 weeks to a month, and in a study of a rat model, it was shown that the internal environment of the wound also returned to normal when the polymer scaffold was degraded within 14 days.^59^ However, the stent in this study can be completely degraded within 12 days, and it's a little shorter than expected in this study. As for the degradation characteristics of the stent, although the degradation time is faster than expected, and the current stent can not meet the clinical needs at present. However, this is only the current degradation characteristics of the stent we studied, which will provide new measures for the future high corrosion resistance of the stent so as to develop a suitable degradation rate to meet the clinical needs of intestinal stent to provide reference.

### 5.3 Current issues associated with magnesium alloy applications in clinical medicine

This study showed that the MAO/PLLA/paclitaxel/Mg–Zn–Y–Nd alloy scaffolds had better corrosion resistance than bare PLLA/Mg–Zn–Y–Nd alloy scaffolds. One of the necessary conditions for good intestinal scaffolds was a stable degradation rate and structural stability during wound healing. When researchers implanted intestinal stents in rats, they found that the wounded tissue began to return to normal after 14 days.^60^ In this study, the stent completely lost its supporting effect after 12 days. Rapid degradation characteristics of the stent were not enough to support the healing of intestinal obstruction, which is related to the more active properties of magnesium. Especially in a complex intestinal environment, the contact between magnesium alloy and intestinal organic and inorganic substances accelerates its degradation rate. However, appropriate degradation rate can be achieved by creating a magnesium alloy stent with a stable structure and sufficient branches during tissue healing. Therefore, it is necessary to further improve the degradable magnesium alloy scaffold to delay its degradation rate. At present, there are many common ways to improve corrosion resistance by changing the alloy surface, such as micro-arc oxidation to form a ceramic layer, impregnation of macromolecule polymers, anodic oxidation treatment, steam treatment, alkali heat treatment, fluoride treatment, ion implantation input, and physical vapor deposition.^61^ However, more research on magnesium alloy degradation rate control is needed. Unremitting efforts have to be made to improve corrosion resistance of magnesium alloy stents in the intestinal environment to support intestinal structure, so that magnesium alloy stents can adapt to the complex and changeable intestinal environment, thus improving clinical treatment of intestinal obstruction diseases.

## 6. Conclusion

In this study, Mg–Zn–Y–Nd alloy filaments were woven into the reticulated scaffolds with inner diameter of 8 mm and length of 20 mm using a monofilament integral braiding method. Magnesium alloy stents exposed to different treatments were implanted into the intestinal tracts of New Zealand white rabbits and the degradation and supporting properties of the intestinal stents coated with paclitaxel were investigated. The results showed that the MAO/PLLA/paclitaxel/Mg–Zn–Y–Nd alloy intestinal stents had better corrosion resistance than the PLLA/Mg–Zn–Y–Nd alloy intestinal stents. When the MAO/PLLA/paclitaxel/Mg–Zn–Y–Nd alloy intestinal stents were implanted into rabbits for 8 to 12 days, stents degraded a great deal *in vivo*, which led to stent structure collapse and discharge from the body. Pathological observations demonstrated that the MAO/PLLA/paclitaxel double-coated drug-eluting Mg–Zn–Y–Nd alloy stents had no significant toxicity to important organs and that the intestinal mucosa around the stent gradually returned to normal within two weeks. There were no significant differences in serological parameters at different degradation stages. The scaffolds also exhibited good biocompatibility. Immunohistochemical evaluation of local intestinal tissue around the stent showed that the PLLA/paclitaxel-coated intestinal stent could inhibit intestinal tissue proliferation. This mechanism provided theoretical support for the future treatment design of intestinal stenosis caused by benign, malignant, and inflammatory hyperplasia.

## Data availability statement

The raw/processed data required to reproduce these findings cannot be shared at this time as the data also forms part of an ongoing study.

## Conflicts of interest

There are no conflicts to declare.

## Supplementary Material
