# Correction: Biological behavior exploration of a paclitaxel-eluting poly-l-lactide-coated Mg–Zn–Y–Nd alloy intestinal stent *in vivo*

**DOI:** 10.1039/d0ra90052d

**Published:** 2020-05-15

**Authors:** Zhanhui Wang, Zongbin Sun, Baowei Han, Qiuxia Zheng, Shaopeng Liu, Bingbing Zhang, Tinghe Duan

**Affiliations:** Department of Surgery, Luoyang Central Hospital Affiliated to Zhengzhou University 288 Zhongzhou Road Luoyang 471000 China zhanhuiwangxyjt@163.com +86 379 6389 2095 +86 379 6389 2095; The Second Affiliated Hospital of Zhengzhou University Zhengzhou 450003 China

## Abstract

Correction for ‘Biological behavior exploration of a paclitaxel-eluting poly-l-lactide-coated Mg–Zn–Y–Nd alloy intestinal stent *in vivo*’ by Zhanhui Wang *et al.*, *RSC Adv.*, 2020, **10**, 15079–15090. DOI: 10.1039/c9ra10156j

The authors regret that incorrect details were given for ref. 52 in the original article. The correct version of ref. 52 is given below as ref. 1.

In the sentence beginning “Stephen *et al.* found that…” on page 15086, the corrected sentence should read as follows: “Shi *et al.* found that a magnesium-based drug delivery system had a stronger long-term inhibitory effect on the proliferation of SMCs cultured *in vitro* compared to stainless steel, which may be related to the degradation of magnesium alloy matrix greatly accelerating and improving the pharmacokinetics of drug release *in vitro*.^52^”

The Royal Society of Chemistry apologises for these errors and any consequent inconvenience to authors and readers.

## Supplementary Material
